# Occurrence and Health Risks of Heavy Metals in Drinking Water of Self-Supplied Wells in Northern China

**DOI:** 10.3390/ijerph191912517

**Published:** 2022-09-30

**Authors:** Miao Bai, Can Zhang, Yuchao Bai, Tianyi Wang, Shaojuan Qu, Hongjuan Qi, Minglu Zhang, Chaohong Tan, Chuanfu Zhang

**Affiliations:** 1 Center for Disease Control and Prevention of Chinese PLA, Beijing 100071, China; 2 Department of Environmental Science and Engineering, Beijing Technology and Business University, Beijing 100048, China; 3 School of Environment and Energy Engineering, Beijing University of Civil Engineering and Architecture, Beijing 102616, China

**Keywords:** self-supplied wells, groundwater, Northern China, heavy metals, health-risk assessment

## Abstract

Self-supplied wells, an important water resource in remote and scattered regions, are commonly deteriorated by environmental pollution and human activity. In this study, 156 self-supplied well-water samples were collected from remote and scattered areas of Inner Mongolia (NMG), Heilongjiang (HLJ), and the suburbs of Beijing (BJ) in Northern China. Twenty-four heavy metals were identified by using the inductively coupled plasma-mass spectrometry (ICP-MS) and inductively coupled plasma-optical emission spectrometry (ICP-OES), and the associated human health risks were assessed by using standards of the US Environmental Protection Agency (US EPA). The concentrations of four heavy metals (As, Fe, Mn, and Tl) in HLJ, one heavy metal (Tl) in BJ, and ten heavy metals (Al, As, B, Cr, Fe, Mn, Mo, Se, Tl, and Zn) in NMG exceeded the limits set by China or the World Health Organization (WHO). The total carcinogenic risk (TCR) and total non-carcinogenic risk (THQ) exceeding set limits mainly occurred in NMG, compared to HLJ and BJ. Moreover, As accounted for 97.87% and 60.06% of the TCR in HLJ and BJ, respectively, while Cr accounted for 70.83% of the TCR in NMG. The TCR caused by Cd in all three areas had a negligible hazard (<10^−4^). As accounted for 51.11%, 32.96%, and 40.88% of the THQ in HLJ, BJ, and NMG, respectively. According to the results of the principal component analysis, heavy metals in well water from HLJ and NMG mainly originated from mixed natural processes and anthropogenic sources, whereas, in BJ, most heavy metals probably originated from natural sources. In the future, long-term monitoring of heavy metals in water from self-supplied wells should be conducted for an extensive range of well-water sites, and well water with high As contamination should be monitored more and fully assessed before being used as a drinking-water source.

## 1. Introduction

Although urban water-supply systems are used in many countries and regions worldwide, some remote and scattered regions or suburban areas still rely on self-supplied wells. For instance, it is estimated that, in 2010, more than 23 million households in the United States used private wells for drinking water [1]. Groundwater accounts for approximately 30% of total global freshwater resources [2], indicating that private wells are an important drinking-water resource in many regions of the world and are essential for human survival. It is estimated that more than 2.5 billion people worldwide rely on groundwater for basic drinking water [3]. The quality of drinking water in self-supplied wells is closely related to the public health of local residents. Well water comes from groundwater and is usually used directly, or after simple treatment, as drinking water. However, groundwater may be contaminated as a result of both naturally occurring sources and human activity, and contaminants such as microorganisms, organic chemicals, fluoride, and heavy metals in well water have been reported to have adverse effects on human health. With economic development, rapid population growth, and urbanization, human activities have negatively affected the quality of groundwater resources. For instance, among the 6124 monitoring wells in China, undrinkable groundwater accounted for 60.1% [4].

Heavy metals are considered one of the major and common pollutants in groundwater [5], having the characteristics of biotoxicity, persistence, and bioaccumulation [6]. Heavy-metal pollutants in groundwater can originate from natural processes and human activity such as mineral weathering, mining activities, agricultural production, industrial manufacturing, and domestic waste stacking [2,7,8]. As, Cd, and Cr are carcinogenic heavy metals that may pose health risks at low concentrations. Among them, As has been identified by the United States Environmental Protection Agency as the only carcinogen that causes cancer in humans through exposure from drinking water [9]. Although some heavy metals are essential elements for human physiological function within a limited concentration, excessive intake can threaten human health [10]. Many researchers have investigated contaminant levels, source identification, and health-risk assessments of heavy metals in groundwater from areas such as the Eastern China coastal zone [11], Majuli River Island [12], north of Kerman province [13], and Northeast Nigeria [14]. However, limited information is available on heavy metals from groundwater in the northern remote and scattered areas of major urban areas in China. In particular, very little information exists on some elements in groundwater, such as Bi, V, and Zr, which are not mentioned in the standards for drinking water of China (SDW) and the World Health Organization (WHO). In this investigation, groundwater from self-supplied well water was collected from the remote and scattered areas of Inner Mongolia (NMG), Heilongjiang (HLJ), and the suburbs of Beijing (BJ) in China. Furthermore, twenty-four kinds of heavy metals that were mentioned or not mentioned in the SDW and WHO standards were studied from groundwater in the suburbs and remote and scattered areas of major northern urban areas in China. The main objectives of this study were to (1) determine the occurrence of heavy metals in well water, (2) use the method recommended by the US Environmental Protection Agency (US EPA) to assess health risks for adults and children, and (3) identify the main sources of heavy metals by using principal component analysis (PCA). This research will contribute to the better management and protection of self-supplied well water, thereby ensuring the safety of drinking water for local inhabitants.

## 2. Material and Methods

### 2.1. Sample Collection

In this study, 156 water samples were collected from May to August 2017 from self-supplied wells in the suburbs and remote and scattered areas of major northern urban areas in China, including HLJ (n = 71), BJ (n = 46), and NMG (n = 39). In the HLJ area, samples were collected from remote and scattered areas of border cities, including Mohe City, Fuyuan City, and Mudanjiang City. Similar to the HLJ, samples were collected in the NMG along the remote and scattered areas from Bayannur City to Hulun Buir City. The remaining samples were collected from the BJ suburbs.

### 2.2. Sampling Preparation and Analysis

Samples were collected in polyethylene bottles. All samples were filtered through a filter membrane with a pore of 0.45 μm, and nitric acid was added to acidify the samples for preservation at pH < 2 to prevent oxidation and bacterial growth. All the samples were stored at 4 °C until further analysis. According to the method recommended by the Chinese Ministry of Environment Protection [15,16], a total of twenty-four heavy metals were evaluated, namely Ag, Al, As, B, Ba, Be, Bi, Cd, Cr, Cu, Fe, Li, Mn, Mo, Ni, Pb, Sb, Se, Sn, Ti, Tl, V, Zn, and Zr. The concentrations of B and Fe were measured by using inductively coupled plasma-optical emission spectrometry (ICP-OES) (Agilent Technologies, 5110, Satan Clara, CA, USA). The remaining elements were analyzed by using inductively coupled plasma-mass spectrometry (ICP–MS) (Agilent Technologies, 7900, Satan Clara, CA, USA). The limits of detection were as follows: Ag (0.03 μg/L), Al (0.60 μg/L), As (0.09 μg/L), B (0.01 mg/L), Ba (0.30 μg/L), Be (0.03 μg/L), Bi (0.03 μg/L), Cd (0.06 μg/L), Cr (0.09 μg/L), Cu (0.09 μg/L), Fe (0.01 mg/L), Li (0.30 μg/L), Mn (0.06 μg/L), Mo (0.06 μg/L), Ni (0.07 μg/L), Pb (0.07 μg/L), Sb (0.07 μg/L), Se (0.09 μg/L), Sn (0.09 μg/L), Ti (0.40 μg/L), Tl (0.01 μg/L), V (0.07 μg/L), Zn (0.80 μg/L), and Zr (0.04 μg/L).

### 2.3. Health-Risk Assessment

In this study, groundwater from the wells was usually used for drinking by local residents in NMG, HLJ, and BJ. Therefore, health risks caused by heavy metals mainly considered the pathway of ingestion, which includes carcinogenic and non-carcinogenic risk. The calculation formulas are as follows [11,17]:

(1) $$CDI_{i}=\frac{C_{i}\times IR\times EF\times ED}{BW\times AT}$$

(2) $$CR_{i}=CDI_{i}\times SF_{i}$$

(3) $$TCR=\overset{n}{\underset{i=1}{\sum }}CR_{i}$$

(4) $$HQ_{i}=\frac{CDI_{i}}{RfD_{i}}$$

(5) $$THQ=\overset{n}{\underset{i=1}{\sum }}HQ_{i}$$

where *CDI_i_* is the chronic daily intake (mg/(kg⋅d)); *CR_i_* is the carcinogenic risk caused by a heavy metal, i; *TCR* is the total carcinogenic risk; *HQ_i_* is the non-carcinogenic risk caused by a heavy metal, i; *THQ* is the total non-carcinogenic risk; *C_i_* is the concentration of a heavy metal, i (mg/L); *IR* is the ingestion rate of water (L/d); *EF* is the exposure frequency (d/a); *ED* is the exposure duration (a); *BW* is the average body weight (kg); *AT* is the average time of exposure (d); *SF_i_* is the cancer slope factor of heavy metal, i ((kg·d)/mg); and *RfD_i_* is the reference dose of heavy metal, i (mg/(kg·d)). The values of *IR*, *EF*, *ED*, *BW*, and *AT* for adults and children are shown in Supplementary Table S1, and those of *SF_i_* and *RfD_i_* are shown in Supplementary Table S2 [11,18,19,20,21].

For the non-carcinogenic risk, if *HQ_i_* or *THQ* < 1, there are no adverse effects on human health, whereas if *HQ_i_* or *THQ* > 1, there may be adverse effects on human health. For the carcinogenic risk, if *CR_i_* or *TCR* < 10^−6^, the carcinogenic risk could be negligible; if 10^−6^ < *CR_i_* or *TCR* < 10^−4^, there is an acceptable carcinogenic risk to humans, and if *CR_i_* or *TCR* > 10^−4^, there may be a high carcinogenic risk to humans [17].

### 2.4. Data Treatment and Statistical Analysis

All statistical analyses were performed by using IBM SPSS Statistics (version 25.0; IBM Corporation, Armonk, NY, USA), with a *p* < 0.05 considered statistically significant.

## 3. Results and Discussion

### 3.1. Characteristics of Heavy Metal Elements in Self-Supplied Wells

#### 3.1.1. Elements Not Mentioned in SDW and WHO Standards

**Figure 1:**
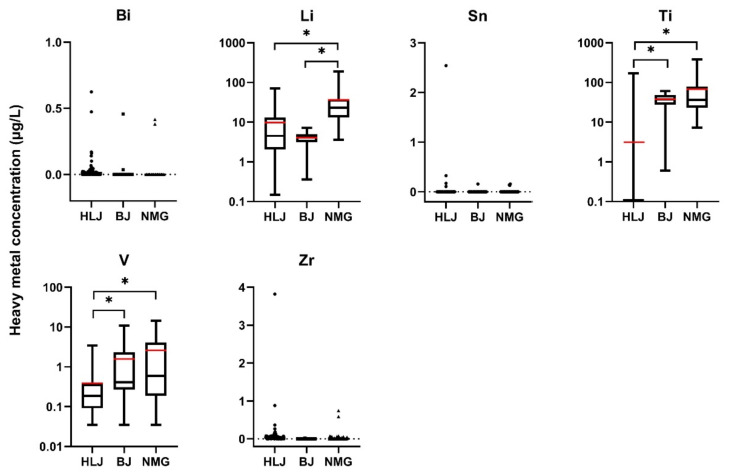
The concentrations of heavy metals in self-supplied wells’ water (elements not mentioned in SDW and WHO). The red line is the average value. * Represents *p* < 0.05.

Figure 1 presents the concentrations of six elements (Bi, Li, Sn, Ti, V, and Zr) in the well water from the three areas. There are no limits available for these six elements in either the SDW or WHO standards. Compared with the orders of magnitude for the concentrations of Li, Ti, and V, the concentrations of Bi, Sn, and Zr were low in all three areas, with 66.03% (103/156), 94.87% (148/156), and 50.64% (79/156) of Bi, Sn, and Zr being not detected (ND).

The average (and maximum) concentrations of Li were 9.80 μg/L (71.33 μg/L), 4.07 μg/L (7.18 μg/L), and 36.39 μg/L (191.50 μg/L) in HLJ, BJ, and NMG, respectively. The Li concentrations in NMG were significantly higher than those in HLJ and BJ (*p* < 0.05). Although more evidence has shown that Li in drinking water may protect against suicide [22], Li exposure through drinking water has been associated with impaired thyroid function in women, including pregnant women, and impaired calcium homeostasis during pregnancy [23,24]. Li levels in Texas public wells ranged between 2.8 and 219 μg/L [22]. In Southeastern Nigeria, the highest Li concentrations in hand-dug wells reached 6.74 μg/L [25]. In domestic supply wells in the United States, Li concentrations ranged from <1 to 1700 μg/L (median of 6 μg/L) [26].

For Ti, 83.10% (59/71) of the concentration in HLJ was ND. The average (and maximum) concentrations of Ti were 3.12 μg/L (170.94 μg/L), 37.76 μg/L (61.07 μg/L), and 67.85 μg/L (379.71 μg/L) in HLJ, BJ, and NMG, respectively. The Ti concentrations in HLJ were significantly lower than those in BJ and NMG (*p* < 0.05). Ti exposure may be associated with several adverse health effects, including diabetes, colitis, cardiopulmonary disorders, and attention-deficit/hyperactivity disorders [27,28]. The Ti concentrations ranged from 0.93 to 3.85 μg/L in Tibet, China [29]. In groundwater from Southern Quebec (Canada), the concentration of Ti reached 0.17 mg/L (170 μg/L) [30]. The median value of Ti the Hetao Plain of China was 4.5 μg/L [18].

The average (and maximum) concentrations of V were 0.39 μg/L (3.47 μg/L), 1.58 μg/L (10.98 μg/L), and 2.60 μg/L (14.36 μg/L) in HLJ, BJ, and NMG, respectively. The V concentrations in HLJ were significantly lower than those in BJ and NMG (*p* < 0.05). It has been reported that V concentrations of approximately >1–10 nM (51–510 ng/L) are toxic to cells, inducing oxidative damage; lipid peroxidation; and changes in the hematological, reproductive, and respiratory systems [31,32]. In Argentina, V concentrations of up to 2.47 mg/L were found in the groundwater of the Southeastern Pampean region [33]. V concentrations in the Hetao Plain of China ranged from 1.05 to 39.30 μg/L, with a median value of 4.37 μg/L [18].

#### 3.1.2. Elements Mentioned in SDW and WHO Standards

(1) Element levels not exceeding the SDW and WHO standards

**Figure 2:**
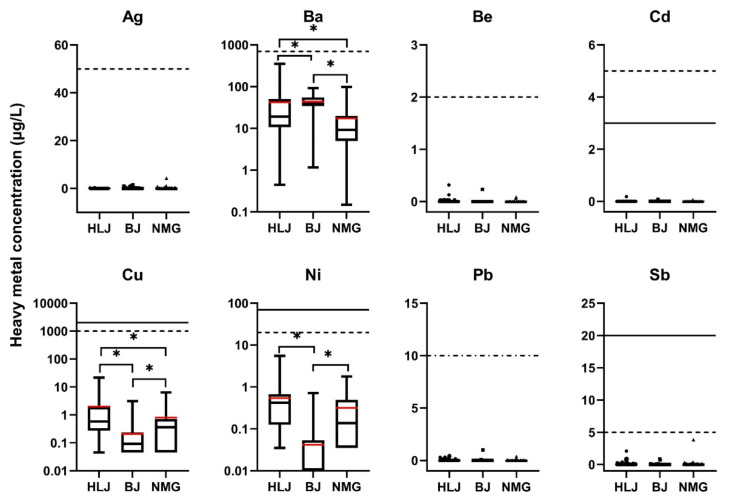
The concentrations of heavy metals in self-supplied wells water (elements mentioned and not exceeding in SDW or WHO). Straight line, WHO; dotted line, SDW; dash-dotted line, SDW and WHO have the same standards limits. The red line is the average value. * Represents *p* < 0.05.

Figure 2 presents the concentrations of eight elements (Ag, Ba, Be, Cd, Cu, Ni, Pb, and Sb) in well water from the three areas; the levels of all eight elements did not exceed their SDW or WHO limits. In all three areas, most of the Ag (76.92%, 120/156), Be (91.03%, 142/156), Cd (98.08%, 153/156), Pb (88.46%, 138/156), and Sb (63.46%, 99/156) were ND. The concentrations of Ba, Cu, and Ni were relatively high in the three areas compared to those of the elements mentioned above (Ag, Be, Cd, Pb, and Sb). The average (and maximum) concentrations of Ba were 42.18 μg/L (353.50 μg/L), 43.82 μg/L (92.02 μg/L), and 17.29 μg/L (98.45 μg/L) in HLJ, BJ, and NMG, respectively. The Ba concentrations in BJ were higher than those in HLJ and NMG (*p* < 0.05). High Ba contamination may be related to cardiovascular and kidney diseases; metabolic, neurological, and mental disorders; and multiple sclerosis and other neurodegenerative diseases [34]. The average (and maximum) concentrations of Cu were 1.99 μg/L (21.80 μg/L), 0.21 μg/L (3.11 μg/L), and 0.80 μg/L (6.46 μg/L) in HLJ, BJ, and NMG, respectively. Higher concentrations of Cu were observed in HLJ than in BJ and NMG (*p* < 0.05). High exposure to Cu in the body causes anemia; high cholesterol; capillary damage; bone changes; and damage to the liver, kidneys, and stomach [35]. For Ni, 73.91% (34/46) of the concentrations in BJ were ND. The average (and maximum) concentrations of Ni were 0.54 μg/L (5.54 μg/L), 0.04 μg/L (0.72 μg/L), and 0.32 μg/L (1.78 μg/L) in HLJ, BJ, and NMG, respectively. The higher concentrations of Ni in HLJ and NMG than in BJ (*p* < 0.05) should be considered because chronic exposure to Ni can cause allergies, DNA damage, neurological disorders, cardiovascular and kidney diseases, lung fibrosis, and lung and nasal cancers [36].

In a previous study, the average concentrations of Ba, Cu, and Ni were 733 μg/L, 19.49 μg/L, and 13.05 μg/L, respectively, for well water in the Chinese Loess Plateau [21]. In drinking groundwater resources of Southeast Iran, Cu concentrations were found to be in a range from 1.07 to 33.18 μg/L, with an average of 9.86 μg/L [13]. In Northeast Nigeria, the median values of Cu and Ni in groundwater were 200 μg/L and 51 μg/L, respectively [14]. In Southern Italy, the median values of Cu and Ni were 11.60 μg/L and 2.30 μg/L, respectively [37].

(2) Elements’ levels exceeding SDW standards

**Figure 3:**
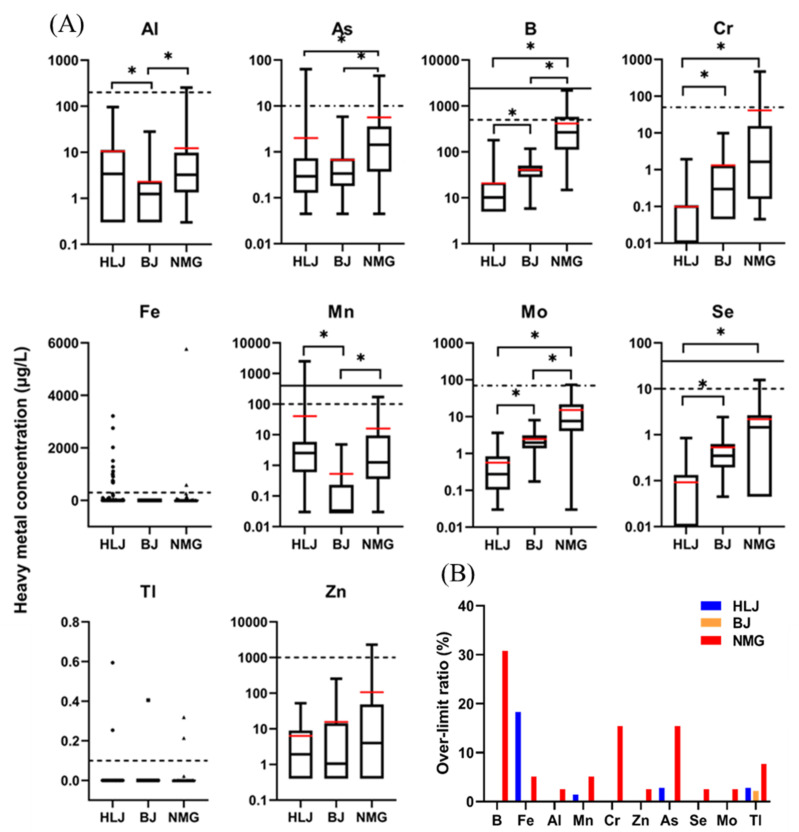
The concentrations of heavy metals in self-supplied wells water. (**A**) Heavy-metals concentration (elements mentioned and exceeding in SDW or WHO). Straight line, WHO; dotted line, SDW; dash-dotted line, SDW and WHO have the same standards limits. The red line is the average value. (**B**) The over-limit ratio of heavy metals. * Represents *p* < 0.05.

Figure 3 presents the concentrations and over-limit ratio of 10 elements (Al, As, B, Cr, Fe, Mn, Mo, Se, Tl, and Zn) in well water from the three areas. In the water wells of HLJ, the concentrations of As, Fe, Mn, and Tl exceeded their SDW limits, and the over-limit ratios were 2.82% (2/71), 18.31% (13/71), 1.41% (2/71), and 2.82% (2/71), respectively (Figure 3B). For HLJ, the average concentrations decreased as follows: Fe (260.18 μg/L) > Mn (40.67 μg/L) > B (20.68 μg/L) > Al (10.53 μg/L) > Zn (6.33 μg/L) > As (2.00 μg/L) > Mo (0.56 μg/L) > Se (0.09 μg/L) > Cr (0.10 μg/L) > Tl (0.01 μg/L) (Figure 3A). For BJ, only Tl exceeded its SDW limit, with an over-limit ratio of 2.17% (1/46) (Figure 3B), and the average concentrations of the heavy metals were ordered as follows: B (40.49 μg/L) > Zn (15.62 μg/L) > Mo (2.46 μg/L) > Al (2.28 μg/L) > Cr (1.31 μg/L) > As (0.67 μg/L) > Mn (0.53 μg/L) ≈Se (0.53 μg/L) > Tl (0.01 μg/L) > Fe (<10 μg/L) (Figure 3A). For NMG, 10 elements exceeded their SDW limits, with the following over-limit ratios: Al (1/39, 2.56%), As (6/39, 15.38%), B (6/39, 30.77%), Cr (6/39, 15.38%), Fe (2/39, 5.13%), Mn (2/39, 5.13%), Mo (1/39, 2.56%), Se (1/39, 2.56%), Tl (3/39, 7.69%), and Zn (1/39, 2.56%) (Figure 3B). Their average concentrations were B (415.74 μg/L) > Fe (182.69 μg/L) > Zn (106.06 μg/L) > Cr (41.07 μg/L) > Mn (15.88 μg/L) > Mo (15.22 μg/L) > Al (12.27 μg/L) > As (5.63 μg/L) > Se (2.18 μg/L) > Tl (0.01 μg/L) (Figure 3A). These results indicated that serious heavy-metal contamination occurred in NMG compared to BJ and HLJ.

The average (and maximum) concentrations of Al were 10.53 μg/L (95.56 μg/L), 2.28 μg/L (28.05 μg/L), and 12.27 μg/L (254.01 μg/L) in HLJ, BJ, and NMG, respectively. The concentrations of Al in BJ were lower than those in HLJ and NMG (*p* < 0.05). For As, the average (and maximum) concentrations were 2.00 μg/L (63.56 μg/L), 0.67 μg/L (5.84 μg/L), and 5.63 μg/L (45.43 μg/L) in HLJ, BJ, and NMG, respectively. NMG had a higher concentration of As than did HLJ and BJ (*p* < 0.05). Moreover, the concentrations of As in some well waters from HLJ and NMG exceeded the SDW limits (10 μg/L). Long-term exposure to As-contaminated drinking water causes increased occurrences of skin, lung, bladder, and kidney cancers and may even result in premature death [38]. In contrast, heavy contamination of B was higher in NMG than in BJ and HLJ (*p* < 0.05). The average (and maximum) concentrations of B were 20.68 μg/L (180.20 μg/L), 40.49 μg/L (117.30 μg/L), and 415.74 μg/L (2174.70 μg/L) in HLJ, BJ, and NMG, respectively. For NMG, the upper quartile of B contamination (576.70 μg/L) exceeded the SDW limits (500 μg/L). Although B has been classified as a non-carcinogenic risk to human health, high concentrations of B affect the reproductive system and cause nervous and metabolic disorders [39,40]. For Cr, 64.79% (46/71) of the well water from HLJ was ND. The average (and maximum) concentrations of Cr were 0.10 μg/L (1.94 μg/L), 1.31 μg/L (9.84 μg/L), and 41.07 μg/L (464.68 μg/L) in HLJ, BJ, and NMG, respectively. The concentrations of Cr in HLJ were significantly lower than those in BJ and NMG (*p* < 0.05). For Fe, 75.64% (118/156) of the concentrations in the three areas were ND. For HLJ and NMG, the average (and maximum) concentrations were 260.18 μg/L (3213.90 μg/L) and 182.69 μg/L (5769.60 μg/L), respectively. The Fe concentrations in some of the wells’ water were higher than their SDW limits (300 μg/L) in HLJ and NMG. In humans, health-related hazards such as hemochromatosis, which results in organ damage, liver cirrhosis, hepatocellular carcinomas, fatigue, joint pain, and hemosiderosis, occur due to high concentrations of Fe in drinking water [41,42,43].

The average (and maximum) concentrations of Mn were 40.67 μg/L (2499.47 μg/L), 0.53 μg/L (4.81 μg/L), and 15.88 μg/L (170.26 μg/L) in HLJ, BJ, and NMG, respectively. The Mn concentrations in BJ were significantly lower than those in HLJ and NMG (*p* < 0.05). For Mo, the average (and maximum) concentrations were 0.56 μg/L (3.62 μg/L), 2.46 μg/L (8.03 μg/L), and 15.22 μg/L (73.08 μg/L) in HLJ, BJ, and NMG, respectively. NMG had the highest Mo concentration, followed by BJ and HLJ (*p* < 0.05). For Se, 70.21% (49/71) of the concentration in HLJ was ND. Its average (and maximum) concentrations were 0.09 μg/L (0.85 μg/L), 0.53 μg/L (2.42 μg/L), and 2.18 μg/L (15.49 μg/L) in HLJ, BJ, and NMG, respectively. In NMG, 28.21% (11/39) of the water wells had higher Se concentrations than the maximum concentrations in HLJ and BJ. The concentration of Se was lower in HLJ than in BJ and NMG (*p* < 0.05). The concentrations of Tl in the majority of the water wells (96.15%, 150/156) were ND in all three areas. However, the maximum concentrations of Tl were 0.59 μg/L, 0.41 μg/L, and 0.32 μg/L in HLJ, BJ, and NMG, respectively, and all exceeded their SDW limits (0.10 μg/L). For Zn, the average (and maximum) concentrations were 6.33 μg/L (52.49 μg/L), 15.62 μg/L (253.79 μg/L), and 106.06 μg/L (2297.49 μg/L) in HLJ, BJ, and NMG, respectively. Furthermore, the maximum concentration of Zn in NMG (2297.49 μg/L) was much higher than its SDW limit (1000 μg/L).

In the Majuli River island of India, the highest concentrations of Fe and Mn were 9040 μg/L and 4480 μg/L, respectively [12], which are much higher than the values presented in this study. The mean concentrations of As, Cr, Fe, and Mn were 41.61 ± 28.18 μg/L, 4.89 ± 3.91 μg/L, 0.084 ± 0.05 mg/L, and 0.010 ± 0.01 mg/L, respectively, in drinking groundwater resources of Rafsanjan City, Kerman, Southeast Iran [13]. The main heavy-metal pollutants in the coastal groundwater of Jiangsu Province in China are B and As, with mean values of 610 μg/L and 20 μg/L, respectively [11]. Moreover, the median concentrations of As, B, Cr, Fe, Mn, Se, Tl, and Zn were 105.66 μg/L, 835 μg/L, 5.6 μg/L, 264.5 μg/L, 105.23 μg/L, 7.04 μg/L, 0.22 μg/L, and 28 μg/L, respectively, in the groundwater of Ischia Island, Southern Italy [37]. For Southern Quebec, Canada, the median concentrations of Al, As, B, Cr, Fe, Mn, Mo, Se, and Zn were 5 μg/L, <1 μg/L, 18 μg/L, <0.5 μg/L, 50 μg/L, 17 μg/L, 0.6 μg/L, <1 μg/L, and 7.5 μg/L in groundwater [30].

### 3.2. Human Health-Risk Assessment

#### 3.2.1. Carcinogenic Risk

**Figure 4:**
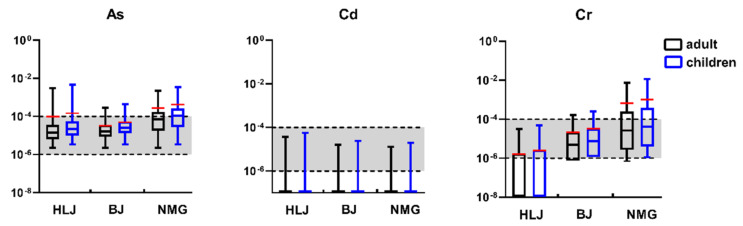
Carcinogenic risk of heavy metals by ingestion of wells water for adults and children. The red line is the average value. The black dotted line is carcinogenic risk values of 10^−6^ or 10^−4^.

Figure 4 presents the carcinogenic risks of three elements (As, Cd, and Cr) associated with the ingestion of well water for adults and children in the three areas. Among the well water of three studied areas, there were no well-water carcinogenic-risk values for As (CR_As_), and the carcinogenic risk was negligible (<1.00 × 10^−6^). The CR_As_ in some wells’ water exceeded the threshold 1.00 × 10^−4^ and was, thus, indicative of a high carcinogenic risk.

For HLJ, the highest CR_As_ for adults (CR_As-adults_) and children (CR_As-children_) were 3.09 × 10^−3^ and 4.75 × 10^−3^, respectively, and 5.63% (4/71) and 8.45% (6/71) of their CR_As_, respectively, exceeded 1.00 × 10^−4^. In BJ, 6.52% (3/46) and 10.87% (5/46) of the CR_As-adults_ and CR_As-children_, respectively, were above the threshold limit of 1.00 × 10^−4^. In the well water of NMG, the average CR_As-adults_ and CR_As-children_ were 2.74 × 10^−4^ and 4.21 × 10^−4^, respectively, which was higher than those of HLJ (9.72 × 10^−5^ and 1.49 × 10^−4^, respectively) and BJ (3.25 × 10^−5^ and 4.99 × 10^−5^, respectively). For CR_As-adults_ and CR_As-children_, up to 43.59% (17/39) and 51.28% (20/39), respectively, showed a cancer risk at CR_As_ > 1.00 × 10^−4^, meaning that no acceptable carcinogenic risk existed for the inhabitants in NMG.

According to the results, 98.08% (153/156) of the water wells showed carcinogenic risk values of Cd (CR_Cd_) < 1.00 × 10^−6^, which means that, in all three areas, the hazard for residents was negligible. Of all the studied samples, 1.92% (3/156) displayed 1.00 × 10^−6^ < CR_Cd_ < 1.00 × 10^−4^, representing an acceptable carcinogenic risk for the water wells. It was concluded that potential carcinogenic health effects of Cd are unlikely to occur through ingestion in HLJ, BJ, and NMG.

For HLJ, the results showed that the carcinogenic risk values of Cr (CR_Cr_) from all the water wells were lower than the maximum acceptable level (1.00 × 10^−4^), meaning that the Cr from these wells had no unacceptable carcinogenic risk. Of these, 33.8% (24/71) and 35.21% (25/71) of CR_Cr_ showed 1.00 × 10^−6^ < CR_Cr_ < 1.00 × 10^−4^ in adults and children, respectively. In BJ, 6.52% (3/46) and 10.87% (5/46) of the CR_Cr_ for adults (CR_Cr-adults_) and children (CR_Cr-children_), respectively, showed a cancer risk of CR > 1.00 × 10^−4^, which indicates that there was no acceptable carcinogenic risk in some water wells. For the CR_Cr-adults_ and CR_Cr-children_, 56.52% (26/46) and 89.13% (41/46) of the studied samples, respectively, displayed 1.00 × 10^−6^ < CR_Cr_ < 1.00 × 10^−4^, which is an acceptable carcinogenic risk, in BJ. In the water wells of NMG, the averages of CR_Cr-adults_ and CR_Cr-children_ were 6.67 × 10^−4^ and 1.02 × 10^−3^, respectively, which were higher than those of HLJ (1.61 × 10^−6^ and 2.47 × 10^−6^, respectively) and BJ (2.13 × 10^−5^ and 3.27 × 10^−5^, respectively). In addition, the highest values of CR_Cr-adults_ and CR_Cr-children_ were 7.54 × 10^−3^ and 1.16 × 10^−2^, respectively, which were far greater than the maximum acceptable level (1.00 × 10^−4^). The CR_Cr-adults_ and CR_Cr-children_ displayed carcinogenic risks higher than the threshold (1.00 × 10^−4^), reaching up to 35.90% (14/39) and 43.59% (17/39), respectively, in NMG. In summary, NMG had higher carcinogenic risks of As and Cr (*p* < 0.05) among the three areas, indicating that more caution is needed when consuming water for local residents. The urine As was found from residents in four villages of Inner Mongolia, indicating that local residents have been exposed to a high As environment for a long-term [44]. It was found that the prevalence of arsenicosis was positively correlated with As intake for those four villages [44]. The findings showed that the number of cancer villages is four, one, and five in HLJ, BJ and NMG by the end of 2017, respectively [45]. Heavy-metal pollution might be a potential driver for villages’ cancer occurrence [45].

#### 3.2.2. Non-Carcinogenic Risk

**Figure 5:**
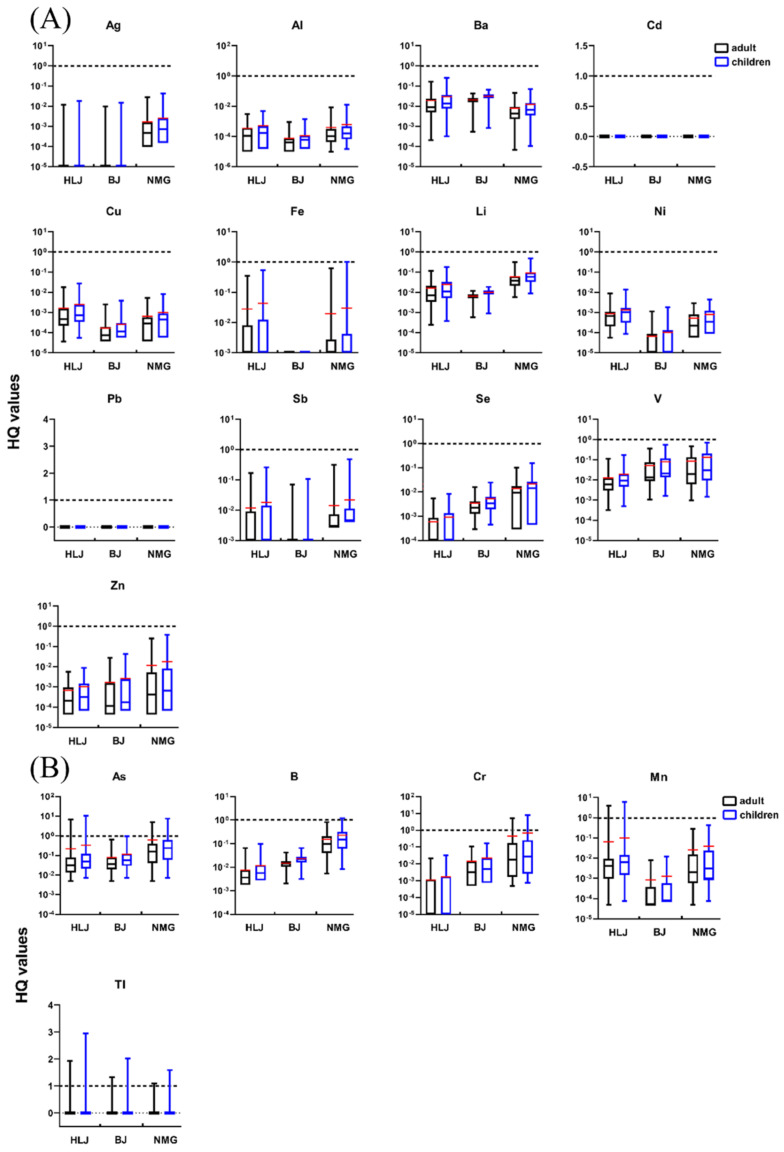
Non-carcinogenic risk of heavy metals by ingestion of wells water for adults and children: (**A**) HQ < 1 and (**B**) HQ > 1. The red line is the average value. The black dotted line is HQ value of 1.

For the non-carcinogenic risks of 13 elements (Ag, Al, Ba, Cd, Cu, Fe, Li, Ni, Pb, Sb, Se, V, and Zn), the HQ values in wells’ water were lower than 1, indicating nonsignificant non-carcinogenic risks (Figure 5A). The order of the average HQs of the heavy metals in the water wells was as follows: for HLJ, Fe > Ba > Li > V > Sb > Cu > Pb > Ni > Zn > Se > Al > Cd > Ag; for BJ, V > Ba > Li> Se > Sb > Zn > Ag > Pb > Cu > Cd > Al > Ni > Fe; for NMG, V > Li > Fe > Sb > Se > Zn > Ba > Ag > Cu > Ni > Al > Pb > Cd.

Figure 5B presents the non-carcinogenic risks of five elements (As, B, Cr, Mn, and Tl) associated with the ingestion of well water for adults and children in the three areas. For all five elements, the values of HQ were above 1 in some water wells, indicating the presence of non-carcinogenic risks possible through ingestion the of well water for adults and children.

For HLJ, 2.82% (2/71), 1.41% (1/71), and 2.82% (2/71) of the HQ values for As (HQ_As_), Mn (HQ_Mn_), and Tl (HQ_Tl_), respectively, were greater than 1 for adults. The highest HQ_As_, HQ_Mn_, and HQ_Tl_ in the adult groups (6.88, 4.06, and 1.93, respectively) were higher than the maximum acceptable level, much higher than those in BJ (0.63, 7.81 × 10^−3^, 1.32, respectively) and NMG (4.92, 2.76 × 10^−1^, 1.04, respectively). In contrast, the HQ values generated for B and Cr were lower than 1 for all water wells in HLJ. For both adults and children in BJ, 2.17% (1/46) of the water wells displayed a high non-carcinogenic risk of exposure to Tl. For several other heavy metals (As, B, Cr, and Mn) in well water from BJ, the HQ values were all below the non-carcinogenic risk threshold limit of 1 for adults and children, confirming the lower risk produced by each heavy metal (As, B, Cr, and Mn) in well water for local residents. In NMG, 17.95% (7/39), 15.38% (6/39), and 2.56% (1/39) of HQ_As_, HQ_Cr_, and HQ_Tl_, respectively, were above the threshold limit of 1. For children in NMG, 20.51% (8/39), 2.56% (1/39), 15.38% (6/39), and 5.13% (2/39) of the HQ_As_, HQ_B_, HQ_Cr_, and HQ_Tl_, respectively, showed high non-carcinogenic risks of HQ > 1. The average HQ_As_, HQ_B_, and HQ_Cr_ for adults in NMG were 6.09 × 10^−1^, 1.50 × 10^−1^, and 4.44 × 10^−1^, respectively, which were higher than for those in HLJ (2.16 × 10^−1^, 7.46 × 10^−3^, and 1.07 × 10^−3^, respectively) and BJ (7.22 × 10^−2^, 1.46 × 10^−2^, and 1.42 × 10^−2^, respectively). Based on the above results, NMG had the highest non-carcinogenic risks for As and B compared to HLJ and BJ (*p* < 0.05). The non-carcinogenic risks of B and Cr in BJ were significantly higher than those in HLJ (*p* < 0.05), and there was a higher non-carcinogenic risk of Mn in HLJ (*p* < 0.05).

#### 3.2.3. Total Health Risk

(1) Total Carcinogenic Risk

**Figure 6:**
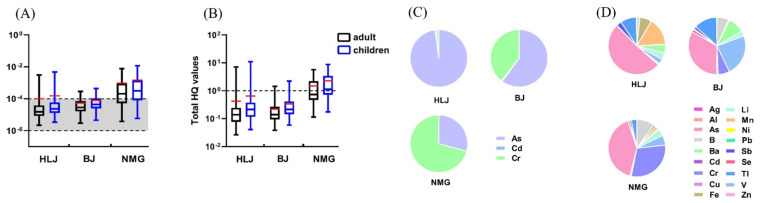
Total health risk and contributions of heavy metals. Total carcinogenic risk (**A**) and total non-carcinogenic risk (**B**). The red line is the average value. The black dotted line is carcinogenic risk values of 10^−6^ or 10^−4^ and HQ value of 1. Contributions of heavy metals in total carcinogenic risk (**C**) and total non-carcinogenic risk (**D**).

A total carcinogenic risk (TCR) > 1.00 × 10^−4^ occurred in some of the water wells, indicating that the TCR posed by carcinogenic heavy metals (As, Cd, and Cr) presented a potential health risk for children and adults in all three areas (Figure 6A and Table 1).

In HLJ, 5.63% (4/71) and 8.45% (6/71) showed TCR >1.00 × 10^−4^ for adults and children, respectively (Figure 6A). The carcinogenic risks generated by As were far greater than those of other carcinogenic heavy metals (Cd and Cr), accounting for 97.87% of the TCR. Cr and Cd accounted for 1.62% and 0.51% of the TCR, respectively (Figure 6C). In BJ, 19.57% (9/46) and 26.09% (12/46) of the TCR were higher than the threshold limit of 1.00 × 10^−4^ for adults and children, respectively (Figure 6A). Similar to that in HLJ, As had the highest proportion in BJ, accounting for 60.06% of the TCR. Cr accounted for 39.30% of the TCR in BJ, whereas Cd accounted for only 0.63% of the TCR in BJ (Figure 6C). For the water wells in NMG, the average TCR for adults and children were 9.41 × 10^−4^ and 1.45 × 10^−3^, respectively, which were higher than those in HLJ (9.93 × 10^−5^ and 1.53 × 10^−4^, respectively) and BJ (5.41 × 10^−5^ and 8.31 × 10^−5^, respectively). The TCR values of carcinogenic heavy metals (As, Cd, and Cr) for adults and children accounted for 66.67% (26/39) and 74.36% (29/39) of the total TRC, respectively, exceeding the limit of 1.00 × 10^−4^, and the rest of the samples had TCR values between 1.00 × 10^−6^ and 1.00 × 10^−4^ (Figure 6A). Among the three carcinogenic elements, Cr contributed the most to the TCR, accounting for 70.83% of the risk generated by all carcinogenic pollutants and is the main heavy metal posing a carcinogenic risk. The carcinogenic risks of As and Cd accounted for 29.14% and 0.04% of the TCR, respectively, both of which were much lower than that of Cr (Figure 6C).

In general, a higher TCR was observed in NMG, followed by BJ, and the lowest values were observed in HLJ (*p* < 0.05). Overall, As contributed the most to the TCR for both adults and children in HLJ and BJ. However, Cr contributed the most to the TCR in NMG. In each of the three areas, the TCR posed by Cd was negligible. Although Cd has strong toxic and carcinogenic effects [11], its low content in all three areas poses a low health risk. However, As in the well water of HLJ and BJ and Cr in the well water of NMG have a high toxicity and high concentration, respectively, resulting in considerable health risks. In a study conducted in Sabzevar, Iran, the carcinogenic risks for both children and adults through ingestion were in order of As > Cr > Cd [7]. In other studies on the carcinogenic risks associated with ingestion, the risk was mainly caused by As and Cr in Dalian, China, and As posed a high risk in Gopalganj in Bangladesh [46,47].

(2) Total non-carcinogenic risk

The total HQ (THQ) values caused by non-carcinogenic heavy metals in some well water for adults and children were higher than 1, which may negatively impact human health in all three areas (Figure 6B and Table 1).

For HLJ, the highest THQ values for adults and children were 7.17 and 11, respectively, which were far more than the HQ threshold value and higher than those in BJ (1.44 and 2.21, respectively) and NMG (5.70 and 8.76, respectively). Furthermore, 7.04% (5/71) of the water wells showed a THQ >1 for both adults and children (Figure 6B). The total non-carcinogenic risks generated by As were far higher than those of other non-carcinogenic heavy metals, accounting for 51.11% of the THQ. The percentage of Mn was 15.61% of THQ (Figure 6D). For adults and children in BJ, 2.17% (1/46) and 4.35% (2/46) of the THQ were beyond the threshold limit of 1, respectively (Figure 6B). Similar to in HLJ, As accounted for 32.96% of the THQ generated by all non-carcinogenic pollutants and is the main heavy metal posing a non-carcinogenic risk. The contributions of V were slightly lower than those of As, ranking second and accounting for 23.46% of the THQ (Figure 6D). For NMG, the averages of THQ values for adults and children were 1.49 and 2.29, respectively, which were far more than those in HLJ (4.23 × 10^−1^ and 6.49 × 10^−1^, respectively) and BJ (2.19 × 10^−1^ and 3.36 × 10^−1^, respectively). The THQ values for adults and children, accounting for 46.15% (18/39) and 53.85% (21/39), respectively, were higher than 1, indicating that non-carcinogenic elements in most of the water wells in NMG can cause health hazards to humans (Figure 6B). Significantly, As in the water wells posed the greatest risk, accounting for 40.88% of the THQ from the non-carcinogenic elements. The contributions of Cr were slightly lower than those of As, accounting for 29.82% of the THQ (Figure 6D).

Overall, the THQ values of the non-carcinogenic heavy metals in NMG were significantly higher than those in HLJ and BJ (*p* < 0.05). Among the non-carcinogenic heavy metals, the non-carcinogenic risk of As was the highest, and As contributed the most to the THQ in the water wells of all three areas. Therefore, the presence of As in water wells may pose lifetime health risks to local residents. Moreover, Mn, V, and Cr also contributed significantly to the non-carcinogenic risks in HLJ, BJ, and NMG, respectively. Meanwhile, the results indicated that, although the HQ values from some individual non-carcinogenic heavy metals in the water wells were below the limit, the THQ values from multiple non-carcinogenic heavy metals may exceed the limit, with children being more susceptible to the effects of heavy-metal pollutants in well water through ingestion. Some studies have shown that As exposure poses a serious non-carcinogenic risk in groundwater from the Hetao Plain in Northern China and Southeast Iran, and this is consistent with our results [13,18].

### 3.3. Possible Sources of Heavy Metals in Wells Water

**Figure 7:**
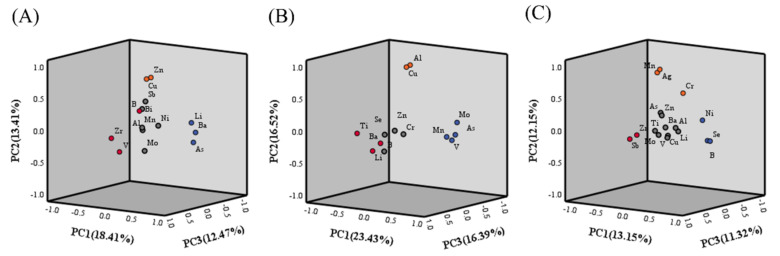
Principal component analysis (PCA) for heavy metals in HLJ (**A**), BJ (**B**), and NMG (**C**).

PCA identified six principal components (PCs) with eigenvalues greater than 1.0, with three key PCs (PC1–PC3) cumulatively explaining 44.29% of the variation in heavy metals in well water from HLJ (Figure 7A). The first PC accounted for 18.41% of the total variance within the dataset and was dominated by As, Ba, and Li, with factor loading values greater than 0.8, which may mainly originate from anthropogenic activities such as mining and smelting of metals, industrial waste discharge, fertilizers, agricultural activities, and wastewater irrigation [48,49]. The second component (PC2) represented 13.41% of the total variance and was dominated by Zn and Cu. Zn is an important raw material for industrial production, particularly for alloy production. Therefore, the presence of these elements in well water may be mainly due to industrial contamination [11]. Variables Zr, B, and V were positively correlated with the third component (PC3), which explained 12.47% of the total variance. Several studies have shown that B has a strong relationship with mineral weathering, agriculture, and mining practices [39].

For heavy metals in well water from BJ, five PCs with eigenvalues greater than 1.0 were extracted, with three key PCs (PC1–PC3) cumulatively explaining 76.24% of the total variance (Figure 7B). PC1 accounted for 23.43% of the total variance, with strong positive loadings of Mo, As, V, and Mn and factor loading values greater than 0.7. These elements may be derived mainly from parent material weathering and pedogenic processes [21,37]. PC2 accounted for 16.52% of the total variance and had strong positive loadings for Al and Cu, both of which may have a lithogenic origin. They may originate from the crust or be formed from carbonate mineral weathering and leaching of the host rock of aquifers [14,21]. PC3 accounted for 16.39% of the total variance and had strong positive loadings for Ti, Ba, and Li. These elements may be predominantly of geogenic origin, derived from multiple primary mineral sources [30,50].

For NMG, the results of the PCA of the heavy-metal content are illustrated in Figure 7C. Eight PCs with eigenvalues greater than 1.0 and three key PCs were extracted and accounted for 36.62% of the total variance. PC1 accounted for 13.15% of the total variance and had the highest loading of Se, followed by B and Ni. Ni may originate from anthropogenic inputs such as industrial waste and coal combustion [21]. Se can be introduced into the environment through natural or anthropogenic sources such as volcanic emanations, coal ore deposits, phosphorites, metal mining, and fossil fuel refinement [51]. PC2 accounted for 12.15% of the variance, with positive loadings of Ag, Cr, and Mn. Mn is often associated with ore, and it has been speculated that the high concentration of Mn in the groundwater is attributed to the weathering and dissolution of an ore containing Mn [11]. PC3 accounted for 11.32% of the variance, with the highest loading rates for Sb and Zr and may have originated from the same source. They are probably found in primary magmatic deposits or are naturally concentrated in sedimentary deposits [52].

According to the above mentioned evidence, heavy metals in well water from HLJ and NMG may have originated from mixed natural processes and anthropogenic sources. Industrialization has always been the pillar of local economic development in the HLJ and NMG, but the industrial structures of the two regions differ. As an old industrial base in China, HLJ started its industrial development earlier, and its industrial structure is dominated by resource-based and heavy industries. In addition, HLJ is one of the major grain-producing areas in China, and its residents engage in various agricultural activities. NMG is rich in mineral and rare-earth resources. NMG is an important energy-oriented region and mineral-resource-based area in China, with an industrial structure dominated by energy, metallurgy, and building materials. In addition, there are many livestock- and poultry-breeding industries in this region. These may cause heavy-metal pollution of different types and sources in the well waters of HLJ and NMG. BJ is the capital of China, and its number and scale of highly polluting industries, such as the livestock, mining, and heavy industries, are significantly lower than those in HLJ and NMG. Therefore, for the BJ suburbs, most heavy metals in well water are likely to originate from natural sources.

## 4. Conclusions

Of the 156 investigated water samples from self-supplied wells in North China, the concentrations of four heavy metal (As, Fe, Mn, and Tl) in HLJ, one heavy metal (Tl) in BJ, and ten heavy metals (Al, As, B, Cr, Fe, Mn, Mo, Se, Tl, and Zn) in NMG exceeded the SDW or WHO limits. Heavy-metal contamination of self-supplied well water was found to be the worst in NMG compared with HLJ and BJ. The average values of the TCR caused by three carcinogenic heavy metals (As, Cd, and Cr) for adults were 9.93 × 10^−5^, 5.41 × 10^−5^, and 9.41 × 10^−4^ in HLJ, BJ, and NMG, respectively, which demonstrated that the TCR in NMG is higher than those in HLJ and BJ (*p* < 0.05). Moreover, As accounted for 97.87% and 60.06% of the TCR in HLJ and BJ, respectively, while Cr in NMG contributed to 70.83% of the TCR. The CR_As_ in HLJ exceeded the threshold limit (>10^−4^), while the CR_As_ and CR_Cr_ exceeded the threshold limits in BJ and NMG. A negligible hazard (<10^−4^) for CR_Cd_ was found in all three areas. The average value of the THQ posed by 18 non-carcinogenic heavy metals for adults were 4.23 × 10^−1^, 2.19 × 10^−1^, and 1.49 in HLJ, BJ, and NMG, respectively, which demonstrated that the THQ in NMG was significantly higher than that in HLJ and BJ (*p* < 0.05). Overall, As contributed the most to THQ in the three areas. Therefore, drinking water with a high As content in the three areas can have significant effects (both carcinogenic and non-carcinogenic) on public health and, thus, requires more attention. The possible sources identified for the heavy metals of HLJ and NMG were natural processes and anthropogenic sources, whereas, in BJ, most heavy metals probably originated from natural sources. Given this, well water with As pollution should be considered when being used as a drinking-water source. We should be undertaking effective measures to purify well water (such as supplementary As removal process), continuously monitor and fully assess the water, prevent further pollution to the water by heavy metals, and provide safe drinking water for local residents.

## Figures and Tables

**Table 1:** Total health risk and highest contributions of heavy metals.

Study Area	TCR			THQ		
	Adult	Children	Heavy Metals	Adult	Children	Heavy Metals
HLJ	2.19 × 10^−6^~3.09 × 10^−3^ (9.93 × 10^−5^)	3.37 × 10^−6^~4.75 × 10^−3^ (1.53 × 10^−4^)	As (97.87%)	2.65 × 10^−2^~7.17 (4.23 × 10^−1^)	4.07 × 10^−2^~11 (6.49 × 10^−1^)	As (51.11%)
BJ	2.92 × 10^−6^~2.85 × 10^−4^ (5.41×10^−5^)	4.49 × 10^−6^~4.38×10^−4^ (8.31 × 10^−5^)	As (60.06%)	3.83 × 10^−2^~1.44 (2.19 × 10^−1^)	5.88 × 10^−2^~2.21 (3.36 × 10^−1^)	As (32.96%)
NMG	3.79 × 10^−6^~7.66 × 10^−3^ (9.41 × 10^−4^)	5.83 × 10^−6^~1.18 × 10^−2^ (1.45 × 10^−3^)	Cr (70.83%)	1.14 × 10^−1^~5.70 (1.49)	1.75 × 10^−1^~8.76 (2.29)	As (40.88%)

## Data Availability

The data that support the findings of this study are available from the corresponding author upon reasonable request.

## Supplementary Materials

The following supporting information can be downloaded at: https://www.mdpi.com/article/10.3390/ijerph191912517/s1, Table S1: Exposure parameters used for health risk assessment. Table S2: *SF_i_* and *RfD_i_* and for the different heavy metals.
