# Supplementary material for: Occurrence and Health Risks of Heavy Metals in Drinking Water of Self-Supplied Wells in Northern China
Source: Int J Environ Res Public Health. 2022 Sep 30;19(19):12517. doi: 10.3390/ijerph191912517 (PMC9566312; doi:10.3390/ijerph191912517)
Supplement: Supplementary file 1 [file ijerph-19-12517-s001.zip › ijerph-1928159-supplementary.pdf]

International Journal of Environmental Research and Public Health

Supplementary Materials

Occurrence and health risks of heavy metals in drinking water of self-supplied wells in northern China

Miao Bai [1,2,†], Can Zhang [1,*], Yuchao Bai [1†], Tianyi Wang [1], Shaojuan Qu [1], Hongjuan Qi [1], Minglu Zhang [2], Chaohong Tan [3] and Chuanfu Zhang [1,*]

[1] Center for Disease Control and Prevention of Chinese PLA, Beijing 100071, China
[2] Department of Environmental Science and Engineering, Beijing Technology and Business University, Beijing 100048, China
[3] School of Environment and Energy Engineering, Beijing University of Civil Engineering and Architecture, Beijing 102616, China
* Correspondence: zhangcancqu@163.com (Can Zhang); hnzcf@126.com (Chuanfu Zhang)
† These authors contributed to this work equally.

Table S1. Exposure parameters used for health risk assessment.

Exposure parameters	Description	Adult	Child	Unit
IR	ingestion rate of water	2.5	0.78	L/d
EF	exposure frequency	350	350	d/a
ED	exposure duration	26	6	a
BW	average body weight	80	15	kg
AT	average time of exposure	8760	2190	d

Table S2. SF_i and RfD_i and for the different heavy metals.

Elements	RfD_i	Elements	RfD_i	Elements	SF_i
Ag	0.005	Li	0.02	As	1.5
Al	1	Mn	0.02	Cd	6.1
As	0.0003	Ni	0.02	Cr	0.5
B	0.09	Pb	0.0014		
Ba	0.07	Sb	0.0004		
Cd	0.0005	Se	0.005		
Cu	0.04	Tl	0.00001		
Cr	0.003	V	0.001		
Fe	0.3	Zn	0.3		
